# Does being a coach benefit clinician-educators? A mixed methods study of faculty self-efficacy, job satisfaction and burnout

**DOI:** 10.1007/s40037-021-00676-7

**Published:** 2021-08-18

**Authors:** Martha J. Elster, Patricia S. O’Sullivan, Virginie Muller-Juge, Leslie Sheu, Sunitha V. Kaiser, Karen E. Hauer

**Affiliations:** 1 Department of Pediatrics, University of California, San Francisco, CA USA; 2 Department of Medicine, University of California, San Francisco School of Medicine, San Francisco, CA USA; 3 Department of Surgery, University of California, San Francisco School of Medicine, San Francisco, CA USA; 5 Departments of Pediatrics and Clinical Epidemiology and Biostatistics, University of California, San Francisco, CA USA

**Keywords:** Burnout, Coaching, Clinician-educators, Job satisfaction, Self-efficacy

## Abstract

**Introduction:**

Coaching is a growing clinician-educator role. Self-efficacy is a powerful faculty motivator that is associated positively with job satisfaction and negatively with burnout. This study examines self-efficacy, job satisfaction, and burnout in coaches and other clinician-educators.

**Methods:**

We conducted a mixed methods study using a quantitative survey followed by qualitative interviews of faculty at the University of California, San Francisco. Coaches (funded 20% full-time equivalents), faculty with other funded education positions (“funded”), and faculty without funded education positions (“unfunded”) completed a 48-item survey addressing self-efficacy (teaching, professional development, and scholarship), job satisfaction, and burnout. Data were analyzed using analysis of variance followed by post-hoc tests and chi-square tests. To elaborate quantitative results, we conducted qualitative interviews of 15 faculty and analyzed data using framework analysis.

**Results:**

202 of 384 faculty (52.6%) responded to the survey; 187 complete surveys were analyzed. Teaching self-efficacy was similar across groups. Coaches and funded educators had significantly higher professional development self-efficacy and job satisfaction than unfunded educators. Burnout was more prevalent in coaches and unfunded educators. Qualitative analysis yielded three themes: sources of reward, academic identity, and strategies to mitigate burnout. Educator roles provide reward that enhances self-efficacy and job satisfaction but also generate competing demands. Coaches cited challenges in forming professional identities and working with struggling learners.

**Discussion:**

The coaching role provides faculty with benefits similar to other funded educator roles, but the particular demands of the coach role may contribute to burnout.

**Supplementary Information:**

The online version of this article (10.1007/s40037-021-00676-7) contains supplementary material, which is available to authorized users.

## Introduction

Clinician-educators are essential to the academic mission. However, they face challenges to career success, including higher clinical demands and less clear paths to promotion than other faculty [1–3]. Clinician-educators must navigate many different roles including advisor, teacher, and mentor and also forge a joint identity as both clinician and educator [4]. They are as a group, therefore, at high risk for burnout and intent to leave academic medicine due to competing demands [5, 6].

Coaching, a role traditional in sports, business, and music, is gaining popularity in medical education as a clinician-educator role [7]. In medical education, a coach is a faculty educator who builds one-on-one, longitudinal learner relationships to motivate change and maximize learner potential.[8] The coach promotes learners’ self-improvement through feedback, self-reflection, and goal setting [8, 9]. Learners working with coaches endorse enhanced comfort with clinical practice, greater self-awareness, and increased receipt of feedback [10–13]. Likewise, coaches experience benefits including professional development, community building, and role satisfaction [11, 13, 14].

In undergraduate medical education, coaches are clinician-educators matched longitudinally with medical students [9]. Coaches, like other clinician-educators, may be asked to serve as teachers, mentors, and advisors but are not involved in formal learner assessment [8, 15, 16]. Many coaching programs provide coaches with protected time, funding, and formal faculty development, similar to other clinician-educator roles [13–15]. The coaching role is unique from other educator roles, however, due to its focus on self-reflection and close learner relationships [9]. At our institution, coaches support students through medical school with a focus on academic performance and professional identity formation. Coach competencies include establishing a trusting relationship, encouraging reflection, and teaching clinical skills [17]. Coaches, using inquiry, guide students to reflect on their performance and set goals. Coaches are expected to model this same approach for their own performance, which differs from other clinician-educator positions [9, 17].

Coaching is therefore a unique faculty role with a self-reflective and performance-focused approach which differs from other clinician-educator roles. However, medical education research at present lacks a clear distinction between coaching and these other roles [16]. Coaching confers benefits to both coaches and learners, but clarity is needed about how the role manifests for educators [4, 8, 16]. We do not know how the coach role differs from other clinician-educator roles or how it contributes to coaches’ experience as educators. Clinician-educators, including coaches, have many responsibilities but must be supported in crafting a professional identity to achieve success and prevent burnout [5]. The present lack of clarity around the coaching role and effects on coaches themselves deserves exploration. Describing the impact of the coach role is important to support coaches in achieving career success and to optimize the clinician-educator experience for all faculty.

Self-efficacy, or judgements of one’s own capabilities, is a powerful faculty motivator and can be applied to the coach experience [18]. Self-efficacy influences individuals’ choices and goals; individuals tend to pursue activities in which they have high self-efficacy. Furthermore, it drives resilience within choices, allowing individuals to see difficulties as challenges rather than obstacles [21]. Clinician-educator self-efficacy encompasses three domains: teaching, professional development, and scholarship [19–21]. Situations which foster clinician-educator self-efficacy include opportunities to practice self-reflection, build skills, and develop community [18, 20]. Self-efficacy is fundamental to faculty career performance as it is associated positively with job satisfaction and negatively with burnout [18, 22–24].

We do not yet know how coaching influences the overall faculty career experience or how it compares with other clinician-educator roles. Our objectives were to examine coaches’ and other educators’ self-efficacy, job satisfaction, and burnout, and then explore how faculty experiences contribute to these outcomes. Coaching is a unique role which encourages self-reflection and offers skill building and community, all of which are potential sources of self-efficacy [14, 16, 25, 26]. We hypothesized that coaches experience higher self-efficacy and, therefore, higher job satisfaction and lower burnout compared with other educators.

## Methods

We conducted a mixed methods study with a sequential explanatory design of clinician-educators including coaches at the University of California, San Francisco (UCSF) School of Medicine [27]. Sequential explanatory design involves two phases: quantitative data collection and analysis followed by qualitative data collection and analysis, with the goal of using the qualitative phase to explain quantitative results.[27–29] We surveyed faculty (Phase 1) and then conducted qualitative interviews (Phase 2) to understand the experience of coaches compared with other educators. A mixed methods approach was selected to quantify self-efficacy, job satisfaction, and burnout among coaches and other clinician-educators and then used interviews to explore faculty experiences based on these findings. The UCSF Institutional Review Board approved this study (19-27651).

### Setting

UCSF is a large, public medical center with multiple clinical teaching sites. The coaching program has 57 physician faculty each paired with approximately 12 medical students to provide longitudinal coaching through medical school. Coaches receive funding (20% full-time equivalents) and participate in regular faculty development, including monthly coach meetings. Outside of coaching, the institution funds clinician-educators in multiple undergraduate and graduate medical education roles. Faculty without education funding engage in direct teaching and all faculty may participate in faculty development.

### Participants

All coaches were invited to participate except for one study investigator (LS) *(n* = 56). For comparison, two groups of non-coach educators were created from mailing lists: faculty with funded education positions who were not coaches (“funded educators”) and faculty without funded education positions (“unfunded educators”). Funded educators were defined as faculty who hold undergraduate and/or graduate medical education roles with salary support (e.g. clerkship site directors, fellowship program directors). Coaches (who also receive salary support) were excluded from this group. Unfunded educators were defined as clinician-educator track faculty who do not receive salary support for education roles but who participate in teaching. Faculty who were ≥ 70% full-time equivalents were included. We selected faculty with similar rank, gender, and department as coaches. Anticipating a lower response among unfunded educators (40%), we aimed for a total of 100 unfunded educators. Altogether, 384 faculty received survey invitations (56 coaches, 69 funded, and 259 unfunded educators). The survey asked faculty to participate in a follow-up interview. Those who agreed were categorized by group and randomly selected for interviews.

### Phase 1: Quantitative analyses

#### Survey development:

We developed a survey to measure self-efficacy, burnout, and job satisfaction, following procedures by Artino, et al. (2014) (please refer to the Electronic Supplementary Material for details) [30]. Survey questions addressed demographics and education roles. Constructs were measured using instruments with validity evidence (below). Nine cognitive interviews were conducted to confirm item understanding; responses were not included in our sample.

#### Teaching self-efficacy:

We used the modified Maastricht Clinical Teaching Questionnaire (mMCTQ) by Bearman, et al. (2018) [31]. The mMCTQ is a 24-item self-assessment tool for clinical preceptors (5-point Likert scale; 1 = “fully disagree”, 5 = “fully agree”) to rate five teaching domains: modeling, coaching, articulation, exploration, and safe learning environment [32, 33]. Mean scores were calculated for each domain and summed items for a total teaching self-efficacy score. Factor analysis supported five domains with Cronbach’s alpha reliability coefficients of 0.55, 0.68, 0.80, 0.86, 0.69, respectively. For domain calculations and factor analysis, we excluded nine items which previous literature demonstrated did not persist in factor analysis [32, 33].

#### Professional development self-efficacy:

We developed a 7-item scale from previously published faculty development surveys (5-point Likert scale; 1 = “weak”, 5 = “strong”) [20, 34, 35] and summed items for a professional development self-efficacy score. Our factor analysis supported a single factor with a Cronbach’s alpha reliability coefficient of 0.85.

#### Scholarship self-efficacy:

We developed a 4-item scale using previously published faculty development surveys (5-point Likert scale; 1 = “weak”, 5 = “strong”) [25, 38, 39] and summed items to create a scholarship self-efficacy score. Using factor analysis, we confirmed a unidimensional factor in our sample with a Cronbach’s alpha reliability coefficient of 0.87.

#### Job satisfaction:

Job satisfaction was assessed using Tyssen’s single-item job satisfaction scale (2013) (5-point Likert scale; 1 = “very dissatisfied”, 5 = “very satisfied”) [36].

#### Burnout:

For burnout, the 2‑item Maslach Burnout Inventory (2009) was used, which rates frequency of burnout symptoms (7-point Likert scale; 0 = “never”, 6 = “every day”). [37–39].

#### Data collection:

Participants completed the survey electronically in Qualtrics (Provo, UT) using a secure, anonymous email invitation. Faculty received up to six reminder emails. Participants received a $ 10 electronic gift card.

#### Statistical analysis:

Descriptive statistics were calculated for faculty demographics. One-way analysis of variance (ANOVA) was used to compare faculty demographics by group for gender and years on faculty. We calculated survey subset scores as means and described means for all faculty groups using standard descriptive statistics. Five score domains were created: teaching self-efficacy, professional development self-efficacy, scholarship self-efficacy, job satisfaction, and burnout. Mean scores were calculated using the Likert scales described above. We used one-way ANOVA to compare means between groups and conducted post-hoc analysis using Student-Newman-Keuls for each domain. For job satisfaction and burnout, the prevalence of high scores was calculated using previously published thresholds. Faculty were categorized as having high job satisfaction if they responded as “satisfied” or higher [36]. We categorized faculty as burned out if they reported symptom frequency as “weekly” or more often for either of the two Maslach Burnout Inventory items [38]. Data were analyzed using SPSS for Windows Version 24 (IBM, Armonk, NY). Significance was set at *p* = 0.01 due to multiple statistical tests.

### Phase 2: Qualitative analyses

#### Interview guide:

Informed by Phase 1 results, we developed an interview guide to enrich our understanding of faculty self-efficacy, job satisfaction, and burnout (see Electronic Supplementary Material) [27, 28]. Questions focused on effective and ineffective coaching, teaching and scholarship, and overall job satisfaction and burnout. Interview questions were designed to explore potential mechanisms driving differential job satisfaction and burnout. We pilot tested the guide with two participants and included the interviews in the analysis.

#### Interviews:

Survey participants were interviewed from all three faculty groups and we continued interviews until sufficient conceptual depth was reached to create connections to Phase 1 results [40]. One author (MJE) conducted interviews from October 2019 to January 2020. Interviews were audio-recorded, professionally transcribed, and de-identified before analysis. Interviewees received a $ 20 electronic gift card.

#### Analysis:

We used a framework method for analysis, a type of thematic analysis defined by use of a matrix to organize and compare codes across participant groups [41]. Authors (MJE, VMJ) used two interviews to develop an initial codebook. All authors reviewed this codebook and agreed upon codes. Authors (MJE, VMJ, LS, KEH) independently coded transcripts using Dedoose Version 8.0.35 software (Los Angeles, CA) with two investigators per transcript and met to reconcile codes and reach consensus. Authors (MJE, VMJ, LS, KEH) analyzed excerpts by code and charted data into a framework matrix, organizing codes by column and faculty group by row. Code interpretations and exemplar quotes were listed in individual cells for comparative analysis.

#### Reflexivity:

Different perspectives were brought to the study. MJE and SVK are pediatric hospitalist physicians, PO’S and VMJ are educational researchers, LS is a coach and KEH is a dean and coaching program director. We discussed how our roles might influence data analysis and used reflective journaling [42].

## Results

### Phase 1: Quantitative results

Overall, 202 of 384 (52.6%) faculty responded to survey requests. Surveys with $\geq$50% items completed were retained; 187 surveys were included in final analysis. Of included respondents, 39 were coaches, 71 funded educators, and 77 unfunded educators (Tab. 1). More survey respondents were female than male (61.5% versus 38.5%) with unfunded educators significantly more male than female (*p* = 0.01).

**Table 1:** Demographics of faculty survey respondents regarding faculty self-efficacy, job satisfaction and burnout at a single institution, 2019

	All faculty	Coaches	Funded educators ^a^	Unfunded educators ^b^
*Respondents: no. (%)*	187/384 (48.7)	39/56 (69.6)	71 ^c^	77 ^c^
*Gender: no. (%)*				
– Female	115 (61.5)	28 (71.8)	50 (70.4)	37 (48.1)
– Male**	72 (38.5)	11 (28.2)	21 (29.6)	40 (51.9)
– Transgender male/female	0 (0)	0 (0)	0 (0)	0 (0)
– Non-binary	0 (0)	0 (0)	0 (0)	0 (0)
*Academic rank: no. (%)*				
– Clinical instructor	2 (1.1)	0 (0)	2 (2.8)	0 (0)
– Assistant professor	69 (36.9)	15 (38.5)	25 (35.2)	29 (37.7)
– Associate professor	64 (34.2)	15 (38.5)	22 (31)	27 (35.1)
– Professor	52 (27.8)	9 (23.1)	22 (31)	21 (27.3)
*Department: no. (%)*				
– Family medicine	9 (4.8)	2 (5.1)	4 (5.6)	3 (3.9)
– Internal medicine	88 (47.1)	20 (51.3)	37 (52.1)	31 (40.3)
– Pediatrics	20 (10.7)	4 (10.3)	5 (7.0)	11 (14.3)
– Surgical fields	32 (17.1)	5 (12.8)	9 (12.7)	18 (23.4)
– Other Specialties	38 (20.3)	8 (20.5)	16 (22.5)	14 (18.2)
*Funded education roles *^d^* : no. (%)*				
– Undergraduate (e.g. clerkship site director)	41 (22.0)	7 (18.0)	34 (47.9)	0 (0)
– Graduate (e.g. fellowship program director)	28 (15.0)	10 (25.6)	18 (25.4)	0 (0)
– Other (e.g. vice chair for education)	3 (1.6)	0 (0)	3 (4.2)	0 (0)
– Multiple roles	17 (9.1)	1 (2.6)	16 (22.5)	0 (0)
Years on faculty: mean (SD)††	8.71 (7.0)	8.26 (5.2)	9.92 (7.6)	7.83 (7.1)

**p = 0.01, ††*p* = 0.18

^a^ Faculty with funded education positions

^b^ Faculty without funded education positions

^c^ Faculty self-identified as either funded or unfunded educators and response rate could not be calculated based on anonymous responses

^d^ Coaching role not included

#### Faculty scores (Tab. 2):

Mean teaching self-efficacy was similar across groups (*p* = 0.17). Coaches and funded educators had significantly higher self-efficacy in establishing a safe learning environment (*p* < 0.01) and had significantly higher professional development self-efficacy (*p* < 0.001) than unfunded educators. Coaches had lower scholarship self-efficacy than funded educators (*p* = 0.03) and did not differ from unfunded educators. Coaches and funded educators reported significantly higher job satisfaction than unfunded educators (*p* = 0.01). Overall, 56.2% of faculty reported burnout. More coaches (64.1%) and unfunded educators (63.2%) experienced burnout than funded educators (44.3%) (*p* = 0.04).

**Table 2:** Self-efficacy, job satisfaction and burnout of faculty survey respondents at a single institution, 2019

Survey domain	All faculty (*n* = 186)	Coaches (*n* = 39)	Funded educators ^a^ (*n* = 71)	Unfunded educators ^b^ (*n* = 76)	*P*‑value
*Self-efficacy domains: mean (SD)*					
– Teaching	4.23 (0.37)	4.28 (0.32)	4.26 (0.38)	4.17 (0.38)	0.17
– Professional Development	3.54 (0.75)	3.63 (0.73)^c^	3.77 (0.72)^c^	3.29 (0.71)^d^	< 0.001
– Scholarship	3.84 (0.93)	3.55 (1)^c^	4.03 (0.82)^d^	3.81 (0.95)^c,d^	0.03
*Teaching self-efficacy sub-domains* [34]*: mean (SD)*					
– Modeling	4.18 (0.54)	4.14 (0.54)	4.16 (0.55)	4.22 (0.48)	0.65
– Coaching	4.19 (0.53)	4.31 (0.37)	4.19 (0.59)	4.14 (0.54)	0.24
– Articulation	4.16 (0.52)	4.19 (0.50)	4.19 (0.54)	4.11 (0.51)	0.63
– Exploration	4.03 (0.72)	4.18 (0.65)	4.11 (0.74)	3.90 (0.71)	0.04
– Safe learning Environment	4.69 (0.37)	4.76 (0.30)^a^	4.76 (0.33)^c^	4.59 (0.41)^d^	< 0.01
*Job satisfaction and burnout: no (%)*					
– Job satisfaction high score ^e^	144 (77.8)	32 (82.1)^c^	63 (90)^c^	49 (64.5)^d^	0.001
– Burnout index, burned out ^f^	104 (56.2)	25 (64.1)^c^	31 (44.3)^d^	48 (63.2)^c^	0.04

^a^ Faculty with funded education positions

^b^ Faculty without funded education positions (1 faculty excluded due to incomplete subscale response)

^c,d^ Significant subset in Student-Newman-Keuls post-hoc analysis (Harmonic Mean Sample Size = 56.5, subset for alpha = 0.05)

^e^ Using a single-item measure of job satisfaction developed by Tyssen R, et al. (2013), with high score defined as “satisfied” or higher

^f^ Using the 2‑item Maslach Burnout Inventory with “burned out” defined by frequency of “weekly” or more often for either item

### Phase 2: Qualitative results

Five interviews were conducted in each faculty group. The 15 interviews averaged 37 min (range: 27–44). Participants were predominantly female (73.3%). We identified three key themes relating to faculty self-efficacy, job satisfaction, and burnout: 1) sources of reward, 2) academic identity and 3) strategies to mitigate burnout. Below we describe themes with representative quotations, participant number, and group (C: coach, F: funded educator, U: unfunded educator) (also see Electronic Supplementary Material).

#### Sources of reward

Faculty described three sources of reward: relationships with learners, learner accomplishments, and their own educator successes. Reward generated both self-efficacy and job satisfaction.

##### Relationships with learners:

Rewarding relationships with learners were generally longitudinal: “*Longitudinal relationships with [learners] are what really brings meaning for me to being an educator.*” (6C) While coaches and funded educators cited opportunities for longitudinal relationships within their roles, unfunded educators reported transient learner relationships in clinical settings. For all groups, trainee engagement was also rewarding, such as when trainees demonstrated excitement about the faculty’s teaching.

##### Learner accomplishments:

Faculty felt reward in seeing learners’ progress during their relationships: “*I try to take joy and satisfaction and feel efficacious in the journey of getting [mentees] towards their destination.*” (2F) Faculty endorsed lack of reward when learners failed to demonstrate progress, such as not responding to feedback.

##### Own educator successes:

All groups felt reward related to their own educator successes, with pride in adapting their teaching skills to different learners and acting as a “guide” for learners developing clinical knowledge. Faculty identified many resources to support their educator successes. Coaches referenced coaching-specific faculty development as especially helpful: “*You’re learning also from your colleagues, not just from students. The different experiences that these faculty educators have had and the incredible breadth of knowledge that they have in these different topics has been so, so helpful*.” (3C) Unfunded educators acknowledged the environment was rich with resources but felt challenged to access them due to competing demands.

#### Academic identity

Faculty described challenges as clinician-educators and, specifically, the unique challenge of how their educator roles fit within a larger professional identity. We identified two subthemes: job composition and challenges to medical education scholarship.

##### Job composition:

Funded roles including coaching provided job diversity and value, which participants found desirable and validating: “*I feel very appreciated and valued for everything I do. And my department values it too*.” (15F) However, coaches and funded educators reported fragmentation of their professional identity through multiple competing roles. Some highlighted a sense of a “patchwork” job and being pulled in multiple directions. Some coaches identified the absence of someone who understood their whole job beyond coaching as unsatisfying: “*I’m really the only person who knows what the heck I’m doing*.” (5C) Coaches felt overwhelmed when their roles required high emotional output to support students with immediate problems: “*I feel like the coaching role is similar to a lot of mentoring roles in that where it does have a cost, there is an emotional output that you have.*” (5C).

##### Challenges to medical education scholarship:

Participants felt dissatisfied in their ability to succeed in medical education scholarship. Barriers included competing demands, lack of mentorship, and lack of time. Some questioned its institutional value: “*I think there is also still a systemic devaluation of medical education scholarship*.” (2F).

#### Strategies to mitigate burnout

Faculty admitted burnout and described coping behaviors. We identified two subthemes: applying cognitive strategies and belonging to an educator community.

##### Applying cognitive strategies:

Faculty recognized but minimized burnout: “*This isn’t a big picture kind of burnout fortunately, just a tiny little one.*” (7U) Burnout prompted self-reflection to gain perspective: “*I’m taking a look and doing some reflective practices about what is and isn’t working, and especially what is and isn’t working as an educator*.” (9F).

##### Belonging to an educator community:

Community served as a source of support through sharing an understanding of experiences. However, the definition of community varied by group. Coaches cited coaching colleagues and leadership as sources of community: “*I have a very good, very small group of coaching colleagues [and] we [have] this informal communication network where we kind of commiserate.*” (5C) Funded educators identified peers and mentors as supports. Unfunded educators identified potential supports but did not cite actively using them.

## Discussion

This study of clinician-educators examines coaches’ and other educators’ self-efficacy, job satisfaction, and burnout, and explores how faculty experiences contribute to these outcomes. Coaches’ experiences were similar to funded educators, with similar professional development self-efficacy, and job satisfaction. However, we noted higher burnout among coaches, whereas unfunded educators experienced both lower job satisfaction and high burnout. Our mixed methods approach allowed us to elaborate that, despite enhanced self-efficacy, coaches experience role tensions which may drive burnout. Coaches detailed emotional output required with struggling learners and a lack of someone who understood their whole job as potential contributors. This study highlights the similarities between coaches and funded educators and identifies challenges for coaches within the larger academic context.

We found that both coaches and funded educators experienced enhanced global job satisfaction. Coaches are known to enjoy role satisfaction, but the finding of the impact of coaching on global satisfaction is novel [10]. Self-efficacy fosters job satisfaction, and participants identified sources of reward that enhanced their self-efficacy [22]. Mastery experiences, or experiences of competency, are a powerful source of self-efficacy [18]. Accordingly, we found that faculty experienced mastery in longitudinal learner relationships and through educator successes. Coaches and funded educators reported significantly higher self-efficacy in creating a safe learning environment which is fundamental to teaching, and likely fosters mastery [43]. Both coaches and funded educators receive some funding for their clinician-educator duties; this support may also explain enhanced job satisfaction. Funding is associated with job satisfaction likely by affording role definition and time [44].

Despite high job satisfaction, almost two-thirds of coaches experienced burnout, a finding which has not been described previously and warrants further study. In interviews, coaches cited struggles with clarifying their professional identity and feeling that no one understood their whole job. Funded educators also cited challenges in forming an academic identity but with less burnout. Clinician-educators with educator roles must reconcile these roles within their professional identity, a task which can be challenging if the roles generate competing demands (role conflict) or if role expectations are unclear (role ambiguity) [45]. Role conflict and ambiguity are associated with burnout and can inhibit professional identity formation [46]. Coaches may be at particular risk for role conflict and ambiguity due to the newness of the role [15, 16]. Coaches also identified emotional resilience required in times of student crisis which could be an additional burnout contributor. Our findings emphasize Watling and LaDonna’s warning against the blurry lines between coaching and other educator roles, and showcase burnout as a potential consequence of this conflict [16]. Thus, coaches and coaching leadership may attend to the risk of burnout and monitor accordingly.

Our findings of professional development self-efficacy and coaching community support previous findings that coaches experience benefits through professional development and a community of practice [14, 26]. Coaches identified coaching peers and leadership as important supports. Supportive work environments enhance faculty work-life balance, a finding echoed by our participants [3]. Our findings that unfunded educators experience challenges accessing resources and less connection to an educator community match literature suggesting they are at risk for burnout [47, 48]. Interviews revealed that unfunded educators may experience fewer longitudinal student relationships which could drive lower satisfaction and burnout.

This study was limited to a single institution and findings may not generalize to all clinician-educators or coaches. Some participants, including coaches, held multiple funded education roles, and thus our three groups are heterogeneous (Tab. 1). Because of this, we could not control for the effects of other roles on the coach experience. Since we drew from existing measures, we did not perform expert survey validation [49]. Our study suggests but cannot prove cause and effect between roles and satisfaction or burnout. Despite intending to explore burnout in depth, faculty minimized burnout, limiting complete exploration. Denial and minimization are known coping strategies for physicians experiencing burnout [50]. We encourage further study among coaches and other clinician-educators.

This mixed methods study explores faculty self-efficacy, job satisfaction and burnout, and elaborates the coaching experience compared with other clinician-educator roles. Coaching appears to confer similar benefits to funded educator roles but may contribute differently to burnout, a finding which needs further exploration. Coaches may face particular challenges given the lack of broader understanding of their role and emotional resilience required with struggling learners [8, 16]. Addressing these challenges may help to strengthen the coach experience and enhance success for coaching programs.

## Supplementary Information

Supplemental materials include 1) Themes and Representative Quotations from Interviews, 2) Survey Instrument, and 3) Interview Guide

## References

[1] Kumar K, Roberts C, Thistlethwaite J (2011). Entering and navigating academic medicine: academic clinician-educators’ experiences. Med Educ.

[2] Levinson W, Rubenstein AB (1999). Mission critical—integrating clinician-educators into academic medical centers. N Engl J Med.

[3] Thomas LR, Roesch J, Haber L (2020). Becoming outstanding educators: what do they say contributed to success?. Adv. Health. Sci. Educ. Theory. Pract..

[4] Stoddard HA, Borges NJ (2015). A typology of teaching roles and relationships for medical education. Med Teach.

[5] Sadowski E, Schrager S (2016). Achieving career satisfaction: personal goal setting and prioritizing for the clinician educator. J Grad Med Educ.

[6] Pololi LH, Krupat E, Civian JT, Ash AS, Brennan RT (2012). Why are a quarter of faculty considering leaving academic medicine? A study of their perceptions of institutional culture and intentions to leave at 26 representative U.S. medical schools. Acad Med.

[7] Lovell B (2018). What do we know about coaching in medical education? A literature review. Med Educ.

[8] Lovell B (2019). Bringing meaning to coaching in medical education. Med Educ.

[9] Deiorio NM, Carney PA, Kahl LE, Bonura EM, Juve AM (2016). Coaching: a new model for academic and career achievement. Med Educ Online.

[10] Iyasere CA, Baggett M, Romano J, Jena A, Mills G, Hunt DP (2016). Beyond continuing medical education: clinical coaching as a tool for ongoing professional development. Acad Med.

[11] Palamara K, Kauffman C, Chang Y (2018). Professional development coaching for residents: results of a 3-year positive psychology coaching intervention. J Gen Intern Med.

[12] de Lasson L, Just E, Stegeager N, Malling B (2016). Professional identity formation in the transition from medical school to working life: a qualitative study of group-coaching courses for junior doctors. BMC Med Educ.

[13] Rassbach CE, Blankenburg R (2018). A novel pediatric residency coaching program: outcomes after one year. Acad Med.

[14] Sheu L, Hauer KE, Schreiner K, van Schaik SM, Chang A, O’Brien BC (2020). “A friendly place to grow as an educator”: a qualitative study of community and relationships among medical student coaches. Acad Med.

[15] Wolff M, Hammoud M, Santen S, Deiorio N, Fix M (2019). Coaching in undergraduate medical education: a national survey. Med Educ Online.

[16] Watling CJ, LaDonna KA (2019). Where philosophy meets culture: exploring how coaches conceptualise their roles. Med Educ.

[17] UCSF Medical Education. Coaching program. 2019. https://meded.ucsf.edu/md-program/current-students/curriculum/bridges-faculty/coaching-program. Accessed 21 Feb 2019.

[18] Pajares F. Self-efficacy beliefs in academic contexts: an outline. 2002. http://des.emory.edu/mfp/efftalk.html. Accessed 21 Feb 2019.

[19] Dybowski C, Kriston L, Harendza S (2016). Psychometric properties of the newly developed physician teaching self-efficacy questionnaire (PTSQ). BMC Med Educ.

[20] Thorndyke LE, Gusic ME, George JH, Quillen DA, Milner RJ (2006). Empowering junior faculty: Penn state’s faculty development and mentoring program. Acad Med.

[21] Sommers PS, Muller JH, Ozer EM, Chu PW (2001). Faculty development: why bother?. Acad Med.

[22] Bandura A (1994). Self-efficacy.

[23] Evers WJG, Brouwers A, Tomic W (2002). Burnout and self-efficacy: a study on teachers’ beliefs when implementing an innovative educational system in the Netherlands. Br J Educ Psychol.

[24] Shoji K, Cieslak R, Smoktunowicz E, Rogala A, Benight CC, Luszczynska A (2016). Associations between job burnout and self-efficacy: a meta-analysis. Anxiety Stress Coping.

[25] Deiorio NM, Skye E, Sheu L (2017). Introduction and definition of academic coaching.

[26] Sekerka LE, Chao J (2003). Peer coaching as a technique to foster professional development in clinical ambulatory settings. J Contin Educ Health Prof.

[27] Creswell JW (2007). Understanding mixed methods research.

[28] Ellaway R (2020). Mixed methods, crimes, and misdemeanours. Adv. Health. Sci. Educ. Theory. Pract..

[29] Fetters MD, Curry LA, Creswell JW (2013). Achieving integration in mixed methods designs-principles and practices. Health Serv Res.

[30] Artino AR, La Rochelle JS, Dezee KJ, Gehlbach H (2014). Developing questionnaires for educational research: AMEE guide no. 87. Med Teach.

[31] Bearman M, Tai J, Kent F, Edouard V, Nestel D, Molloy E (2018). What should we teach the teachers? Identifying the learning priorities of clinical supervisors. Adv in Health Sci Educ.

[32] Boerboom TBB, Dolmans DHJM, Jaarsma ADC, Muijtjens AMM, Van Beukelen P, Scherpbier AJJA (2011). Exploring the validity and reliability of a questionnaire for evaluating veterinary clinical teachers’ supervisory skills during clinical rotations. Med Teach.

[33] Rodino AM, Wolcott MD (2019). Assessing preceptor use of cognitive apprenticeship: is the Maastricht clinical teaching questionnaire (MCTQ) a useful approach?. Teach Learn Med.

[34] Feldman MD, Arean PA, Marshall SJ, Lovett M, O’Sullivan P (2010). Does mentoring matter: results from a survey of faculty mentees at a large health sciences university. Med Educ Online.

[35] Garman KA, Wingard DL, Reznik V (2001). Faculty development: why bother?. Acad Med.

[36] Tyssen R, Palmer KS, Solberg IB, Voltmer E, Frank E (2013). Physicians’ perceptions of quality of care, professional autonomy, and job satisfaction in Canada, Norway, and the United States. BMC Health Serv Res.

[37] West CP, Dyrbye LN, Satele DV, Sloan JA, Shanafelt TD (2012). Concurrent validity of single-item measures of emotional exhaustion and depersonalization in burnout assessment. J Gen Intern Med.

[38] West CP, Dyrbye LN, Sloan JA, Shanafelt TD (2009). Single item measures of emotional exhaustion and depersonalization are useful for assessing burnout in medical professionals. J Gen Intern Med.

[39] Dyrbye LN, West CP, Satele D (2014). Burnout among U.S. medical students, residents, and early career physicians relative to the general U.S. population. Acad Med.

[40] Nelson J (2017). Using conceptual depth criteria: addressing the challenge of reaching saturation in qualitative research. Qual Res.

[41] Gale NK, Heath G, Cameron E, Rashid S, Redwood S (2013). Using the framework method for the analysis of qualitative data in multi-disciplinary health research. BMC Med Res Methodol.

[42] Ortlipp M (2008). Keeping and using reflective journals in the qualitative research process. Qual Rep.

[43] Stalmeijer RE, Dolmans DHJM, Wolfhagen IHAP, Muijtjens AMM, Scherpbier AJJA (2010). The Maastricht clinical teaching questionnaire (MCTQ) as a valid and reliable instrument for the evaluation of clinical teachers. Acad Med.

[44] Demmy TL, Kivlahan C, Stone TT, Teague L, Sapienza P (2002). Physicians’ perceptions of institutional and leadership factors influencing their job satisfaction at one academic medical center. Acad Med.

[45] Bruce SP (2009). Recognizing stress and avoiding burnout. Curr Pharm Teach Learn.

[46] Nassar AK, Waheed A, Tuma F (2019). Academic clinicians’ workload challenges and burnout analysis. Cureus.

[47] Hauer KE, Papadakis MA (2010). Assessment of the contributions of clinician educators. J Gen Intern Med.

[48] Aronsson G (2017). A systematic review including meta-analysis of work environment and burnout symptoms. BMC Public Health.

[49] Magee C, Rickards G, Byars AL, Artino AR (2013). Tracing the steps of survey design: a graduate medical education research example. J Grad Med Educ.

[50] Doolittle BR, Windish DM, Seelig CB (2013). Burnout, coping, and spirituality among internal medicine resident physicians. J Grad Med Educ.

