# Supplementary material for: Does being a coach benefit clinician-educators? A mixed methods study of faculty self-efficacy, job satisfaction and burnout
Source: Perspect Med Educ. 2021 Aug 18;11(1):45–52. doi: 10.1007/s40037-021-00676-7 (PMC8371581; doi:10.1007/s40037-021-00676-7)
Supplement: Supplementary file 1 — Supplemental materials include 1) Themes and Representative Quotations from Interviews, 2) Survey Instrument, and 3) Interview Guide [file 40037_2021_676_MOESM1_ESM.docx]

Exhibit 1: Themes and Representative Quotations from Interviews with 15 Faculty Educators Regarding Self-Efficacy, Job Satisfaction, and Burnout, 2019-2020

| Theme | Description | Representative Quote(s)* |
| --- | --- | --- |
| **Sources of Reward** | | |
| Relationships with Learners | Relationships with learners, particularly longitudinal relationships, generate reward. | “I think that there is so much more as an educator. There’s so much more than just the hour-long teaching sessions or the five-minute precepting and that really what matters to me as an educator and [what] helps with my resilience as an educator is the personal relationships.” (6C)  “I think that’s one of the reasons I stay at [the institution], because I have those longitudinal relationships. It’s not the patient care that keeps me here, and I love seeing patients, but I can see patients anywhere. But these relationships are really different.” (10C) |
| Learner Accomplishments | Watching trainees’ progress over time generates faculty self-efficacy and satisfaction. | “For me, I really feel happy and satisfied and rewarded when I see other people being successful, and it makes me feel good that I helped someone become successful.” (2F) |
| Own Educator Successes | Faculty’s own intrinsic teaching skills and acting as a “guide” for learners generates reward. Faculty development also helps bolster this reward. | “I felt effective in the way that I was able to demonstrate my passion for the topic and why… I wanted to teach that topic to them and was able to kind of engage them beyond just the content.” (6C)  “I learned that when you are really excited about a topic [it] makes you… invested in it and you want to present it in an interesting way so other people feel invested in it. That can be a successful strategy.” (7U) |
| **Academic Identity** | | |
| Job Composition | Funded education roles, including coaching, are desired as they give educator activities value and credit. However, these roles can lead to competing priorities and can make creating a singular academic identity challenging. Some coaches identified working with struggling learners within their role as a potential contributor to burnout. | “I feel like people are saying all the time how much they value what I do and then I get financial compensation for that which is great. So yes, I would say I feel very appreciated and valued for everything I do. And my department values it too.” (15F)  “I feel blessed to have and appreciate the faculty support I get for coaching. That’s when I know time matters because when I do have protected time, I can go all in on really giving the students those several hours of uninterrupted teaching and preparation.” (14C)  “Because my [funded roles] are different, they’re in very different realms. Most people who have a job have one main job that they work on. And I think [in] academic medicine our jobs are kind of… like a patchwork of different bosses for different work… I feel like there are a lot of different expectations but also [every] piece of your job has an email component to it.” (5C)  “There can be times where things hit the fan, and maybe that there's a student crisis going on that week, or that day, and you have a lot of other things that you need to get done.” (3C) |
| Challenges to Medical Education Scholarship | Faculty expressed tension around their ability to be successful in medical education scholarship and some questioned its institutional value. | “I feel like as a clinician-educator, it’s just very different. I am getting paid primarily by my clinical job… Educational research becomes something that I do – either I make the conscious decision that today I’m not going to do these clinical things, I’m going to focus on the research, or it becomes what I work on between the time my kids go to bed and before I go to sleep. Sleep will suffer. It’s not just me. That is how it is for a clinician-educator.” (12F)  “Some of [the inefficacy] is… an individual challenge of time management and… some of that is a system challenge of reimbursing faculty for engaging in scholarship, when really what we’re paid for as medical educators is time in front of students or learners.” (5C) |
| **Strategies to Mitigate Burnout** | | |
| Cognitive Strategies | Faculty acknowledged but tended to minimize their experience of burnout. Burnout often prompted self-reflection followed by the identification of self-care strategies. | “Perspective I think is helpful… for me, at least, these feelings are usually temporary.” (7U)  “One thing I’ve decided is I’m not taking any clinical work back home. As in, if it’s not done, it’s not done. It’s just going to have to wait, but I’m going to go back home and I’m going to sit with my family.” (11U) |
| Belonging to an Educator Community | Community is a support for faculty in times of burnout. Coaches use both coaching peers and leadership. | “Other coaches made me feel a little better, because a lot of coaches are having similar experiences and so [being] able to talk about it out loud and able to get some pointers … I think was very helpful.” (3C)  “The way I typically manage those overwhelming experiences is talking about it to peers… Just hearing about their experiences, both good and bad, could be helpful.” (4F) |

*Illustrative quotes provided with interviewee number and faculty group (C=coach, F=funded educator, U=unfunded educator)

Exhibit 2: Survey Instrument Measuring Faculty Self-Efficacy, Job Satisfaction, and Burnout, 2019

1. How many years have you been on faculty?

_________

1. What is your current academic rank?

[ ] Clinical Instructor

[ ] Assistant Professor

[ ] Associate Professor

[ ] Professor

1. What is your series?

[ ] Clinical X

[ ] Health Sciences Clinical

[ ] Other (please specify): ____________

[ ] Don’t know

1. Describe the division of time that you allot (as FTE or stipend) to specific duties in your current position (must add to 100%)

_____% Clinical

_____% Research

_____% Education (including direct teaching, education leadership)

_____% Administration (including non-education leadership or administration role)

_____% Other (please specify):__________

1. In your teaching role, how would you describe your learners?

[ ] Only undergraduate medical students

[ ] Only residents/fellows

[ ] Primarily undergraduate medical students

[ ] Primarily residents/fellows

[ ] Equal undergraduate medical students and residents/fellows

[ ] Other (please specify): ___________

1. Are you a Bridges coach in the School of Medicine Coaching Program?

[ ] Yes

[ ] No

1. What educational administration roles, if any, do you currently hold? (Check all that apply)

[ ] UME Course Director or Co-Director

[ ] UME Clerkship Leadership

[ ] GME Leadership

[ ] Dean, Associate Dean, or Vice Chancellor

[ ] Other (please specify): _____________

1. Which category most closely describes your department?

[ ] Family Medicine

[ ] Internal Medicine

[ ] Pediatrics

[ ] Surgical Field (e.g. general surgery, neurosurgery, OBGYN, ophthalmology, orthopedics, otolaryngology, plastic surgery, urology)

[ ] Other Specialty (e.g. anesthesiology, dermatology, emergency medicine, genetics, neurology, pathology, psychiatry, radiation oncology, radiology)

1. What is your current gender identity?

[ ] Male

[ ] Female

[ ] Transgender Male

[ ] Transgender Female

[ ] Non-binary

[ ] Something else (please specify): ____________

Please indicate your level of agreement with the following statements regarding teaching medical students, residents, and/or fellows.

1. I consistently demonstrate how to perform clinical tasks.

[ ] Fully Disagree

[ ] Disagree

[ ] Neutral

[ ] Agree

[ ] Fully Agree

1. I clearly explain the most important element for the execution of a given task.

[ ] Fully Disagree

[ ] Disagree

[ ] Neutral

[ ] Agree

[ ] Fully Agree

1. I create sufficient opportunity for the learner to observe me.

[ ] Fully Disagree

[ ] Disagree

[ ] Neutral

[ ] Agree

[ ] Fully Agree

1. I serve as a role model as to the kind of health professional learners would like to become.

[ ] Fully Disagree

[ ] Disagree

[ ] Neutral

[ ] Agree

[ ] Fully Agree

1. I observe learners multiple times during patient encounters.

[ ] Fully Disagree

[ ] Disagree

[ ] Neutral

[ ] Agree

[ ] Fully Agree

1. I give useful feedback during or immediately after direct observation of the learner’s encounters.

[ ] Fully Disagree

[ ] Disagree

[ ] Neutral

[ ] Agree

[ ] Fully Agree

1. I help the learner understand which aspects they need to improve.

[ ] Fully Disagree

[ ] Disagree

[ ] Neutral

[ ] Agree

[ ] Fully Agree

1. I adjust my teaching activities to the level of experience of learners.

[ ] Fully Disagree

[ ] Disagree

[ ] Neutral

[ ] Agree

[ ] Fully Agree

1. I offer sufficient opportunities to learners to perform activities independently.

[ ] Fully Disagree

[ ] Disagree

[ ] Neutral

[ ] Agree

[ ] Fully Agree

1. I support learners in activities that they find difficult to perform.

[ ] Fully Disagree

[ ] Disagree

[ ] Neutral

[ ] Agree

[ ] Fully Agree

1. I gradually reduce the support given, to allow learners to perform certain activities more independently.

[ ] Fully Disagree

[ ] Disagree

[ ] Neutral

[ ] Agree

[ ] Fully Agree

1. I ask learners to provide a rationale for their actions.

[ ] Fully Disagree

[ ] Disagree

[ ] Neutral

[ ] Agree

[ ] Fully Agree

1. I help learners become aware of gaps in their knowledge and skills.

[ ] Fully Disagree

[ ] Disagree

[ ] Neutral

[ ] Agree

[ ] Fully Agree

1. I ask learners questions aimed at increasing their understanding.

[ ] Fully Disagree

[ ] Disagree

[ ] Neutral

[ ] Agree

[ ] Fully Agree

1. I encourage learners to ask me questions to increase their understanding.

[ ] Fully Disagree

[ ] Disagree

[ ] Neutral

[ ] Agree

[ ] Fully Agree

1. I stimulate learners to explore their strengths and weaknesses.

[ ] Fully Disagree

[ ] Disagree

[ ] Neutral

[ ] Agree

[ ] Fully Agree

1. I stimulate learners to consider how they could improve their strengths and weaknesses.

[ ] Fully Disagree

[ ] Disagree

[ ] Neutral

[ ] Agree

[ ] Fully Agree

1. I encourage learners to formulate learning goals.

[ ] Fully Disagree

[ ] Disagree

[ ] Neutral

[ ] Agree

[ ] Fully Agree

1. I encourage learners to pursue their learning goals.

[ ] Fully Disagree

[ ] Disagree

[ ] Neutral

[ ] Agree

[ ] Fully Agree

1. I encourage learners to learn new things.

[ ] Fully Disagree

[ ] Disagree

[ ] Neutral

[ ] Agree

[ ] Fully Agree

1. I create a safe learning environment.

[ ] Fully Disagree

[ ] Disagree

[ ] Neutral

[ ] Agree

[ ] Fully Agree

1. I take sufficient time to supervise learners.

[ ] Fully Disagree

[ ] Disagree

[ ] Neutral

[ ] Agree

[ ] Fully Agree

1. I am genuinely interested in the learners.

[ ] Fully Disagree

[ ] Disagree

[ ] Neutral

[ ] Agree

[ ] Fully Agree

1. I show respect to learners.

[ ] Fully Disagree

[ ] Disagree

[ ] Neutral

[ ] Agree

[ ] Fully Agree

1. I develop a sense of community among learners from diverse backgrounds.

[ ] Fully Disagree

[ ] Disagree

[ ] Neutral

[ ] Agree

[ ] Fully Agree

Please rate your level of confidence from **weak to strong** in your proficiency regarding the following scholarship skills:

1. Writing an article for publication

[ ] Weak

[ ] -

[ ] Neutral

[ ] -

[ ] Strong

1. Writing an abstract for a conference

[ ] Weak

[ ] -

[ ] Neutral

[ ] -

[ ] Strong

1. Participating as part of a scholarly project team

[ ] Weak

[ ] -

[ ] Neutral

[ ] -

[ ] Strong

1. Mentoring learners (medical students, residents, fellows) in scholarly projects

[ ] Weak

[ ] -

[ ] Neutral

[ ] -

[ ] Strong

Please rate your level of confidence from **weak to strong** in your proficiency regarding the following professional development skills:

1. Identifying your professional goals and interests

[ ] Weak

[ ] -

[ ] Neutral

[ ] -

[ ] Strong

1. Identifying the requirements for advancement and promotion in your series at UCSF

[ ] Weak

[ ] -

[ ] Neutral

[ ] -

[ ] Strong

1. Seeking opportunities to collaborate across your own professional network

[ ] Weak

[ ] -

[ ] Neutral

[ ] -

[ ] Strong

1. Maintaining peer networks within UCSF

[ ] Weak

[ ] -

[ ] Neutral

[ ] -

[ ] Strong

1. Maintaining peer networks outside of UCSF

[ ] Weak

[ ] -

[ ] Neutral

[ ] -

[ ] Strong

1. Selecting an appropriate person as your mentor

[ ] Weak

[ ] -

[ ] Neutral

[ ] -

[ ] Strong

1. Approaching institutional leadership

[ ] Weak

[ ] -

[ ] Neutral

[ ] -

[ ] Strong

Please complete the following questions regarding job satisfaction and burnout.

1. On the whole, how satisfied are you with your job?

[ ] Very dissatisfied

[ ] Somewhat dissatisfied

[ ] Neither satisfied nor dissatisfied

[ ] Somewhat satisfied

[ ] Very satisfied

1. I feel burned out from my work.

[ ] Never

[ ] A few times a year or less

[ ] Once a month or less

[ ] A few times a month

[ ] Once a week

[ ] A few times a week

[ ] Every day

1. I have become more callous toward people since I took this job.

[ ] Never

[ ] A few times a year or less

[ ] Once a month or less

[ ] A few times a month

[ ] Once a week

[ ] A few times a week

[ ] Every day

Exhibit 3: Interview Guide from Interviews with 15 Faculty Educators Regarding Self-Efficacy, Job Satisfaction, and Burnout, 2019-2020

Thank you for agreeing to participate in this interview. We appreciate your time and willingness to share your experiences with us. The purpose of this study is to follow up a recent faculty survey you completed in order to understand the experience of faculty at UCSF and faculty self-efficacy. Self-efficacy means an individual’s self-beliefs or judgements of their own capabilities.

There will be three sections to this interview: your experience with teaching and mentoring, your experience with scholarship, and your experience with job satisfaction and burnout. Please try to be specific and think about your experiences as an educator, rather than your clinical experiences, if you can. I would like to record this interview, and then have it transcribed, if that is okay. Your name and any identifying information will be removed during transcription and not used during analysis. You may stop the interview at any time. Do you have any questions?

*Recorder on*

This is an interview by Martha Elster with (Study ID). Today is (date).

The first set of questions will focus on your self-efficacy as an educator and I would like you to first specifically think about your experiences as a mentor or a coach to learners.

1. Over the past year, can you tell me about a time that you felt effective as a mentor/coach? I’ll give you a minute to think of a specific example.
   1. Could you walk me through the experience?
   2. What made you feel you could do this?
   3. Did you talk with anyone for help or advice with this situation?
   4. Why do you think you felt effective in this situation? What contributed to that feeling?
   5. Was there anything more that could have helped you with this?
2. Over the past year, can you tell me about a time that you felt ineffective as a mentor/coach? I’ll give you a minute to think of a specific example.
   1. Could you walk me through how you handled the situation?
   2. Beforehand, did you feel you could accomplish this? What made you feel you could not do this?
   3. Did you talk with anyone for help or advice with this situation?
   4. Why do you think you felt ineffective in this situation? What contributed to that feeling?
   5. Was there anything more that could have helped you with this?

Now I’d like you to think of another example but would like you to think about your role as a teacher to learners.

1. Could you tell me about a time over the past year which you felt effective as a teacher?
   1. Could you walk me through the experience?
   2. Why do you think you were successful in this experience?
   3. What helped you be successful in this experience?
2. Could you tell me about a time over the past year which you felt ineffective as a teacher?
   1. Could you walk me through how you handled the situation?
   2. Why do you think you were unsuccessful in this experience?
   3. What barriers exist which might keep you from feeling more successful in your teaching role?

The second set of questions will focus on your role as an educator and your experience with scholarship. I know that there is a wide range in the amount of scholarship educators like yourself might have or not had so if you could please answer the questions to the best of your ability. Scholarship is defined as activities such as writing abstracts, writing manuscripts, or mentoring learners or peers in research projects.

1. Over the past year, could you describe a time you have felt effective as an educator in scholarship?
   1. Why do you think you felt that way?
   2. What factors contributed to this feeling?
   3. What factors facilitate your ability to engage in scholarship to the extent that you’d like to?
2. Over the past year, could you describe a time you have felt ineffective as an educator in scholarship?
   1. Why do you think you felt that way?
   2. What factors contributed to this feeling?
   3. What are barriers to your ability to engage in scholarship to the extent that you’d like to?

The last set of questions will focus on your experience with emotions you may have experienced as an educator. I’ve asked about specific parts of your job, now I’m hoping to talk about your overall job. I will ask you to please think of examples outside of patient care or the clinical realm if possible.

1. Over the past year, could you describe a time when you felt really satisfied as an educator?
   1. Why do you think you felt this way?
   2. What did you learn from this situation?
   3. How have your funded roles as an educator contributed to your job satisfaction overall?
2. Over the past year, could you describe time when you felt really dissatisfied as an educator?
   1. Why do you think you felt this way?
   2. What did you learn from this situation?
   3. How have your funded roles as an educator contributed to any job dissatisfaction you might have experienced?

I will now ask one question about burnout, but before I do, I want to acknowledge that burnout is a common feeling many of us as physicians experience. I want to thank you for taking time to share your experiences with me. I will again ask you to think beyond your clinical role and share with me experiences as an educator.

1. Could you describe a time when you had feelings of burnout as an educator?
   1. Why do you think you felt this way?
   2. What helps you to manage when you feel this way?
   3. How might your role as educator/coach influence your experience of burnout?

Thank you very much for your thoughts and for sharing with me today. What last thoughts about your role as coach or educator would you like to share with me?
